# A High-Precision Vehicle Detection and Tracking Method Based on the Attention Mechanism

**DOI:** 10.3390/s23020724

**Published:** 2023-01-08

**Authors:** Jiandong Wang, Yahui Dong, Shuangrui Zhao, Zhiwei Zhang

**Affiliations:** School of Computer Science and Technology, Xidian University, Xi’an 710071, China

**Keywords:** vehicle detection, vehicle tracking, attention mechanism, data association

## Abstract

Vehicle detection and tracking technology plays an important role in intelligent transportation management and control systems. This paper proposes a novel vehicle detection and tracking method for small target vehicles to achieve high detection and tracking accuracy based on the attention mechanism. We first develop a new vehicle detection model (YOLOv5-NAM) by adding the normalization-based attention module (NAM) to the classical YOLOv5s model. By exploiting the YOLOv5-NAM model as the vehicle detector, we then propose a real-time small target vehicle tracking method (JDE-YN), where the feature extraction process is embedded in the prediction head for joint training. Finally, we present extensive experimental results to verify our method on the UA-DETRAC dataset and to demonstrate that the method can effectively detect small target vehicles in real time. It is shown that compared with the original YOLOv5s model, the mAP value of the YOLOv5-NAM vehicle detection model is improved by 1.6%, while the MOTA value of the JDE-YN method improved by 0.9% compared with the original JDE method.

## 1. Introduction

With the rapid development of intelligent transportation systems, the explosive growth of vehicular communication services has led to the shortage of radio spectrum resources for vehicular network communications [1,2]. Cognitive radio-enabled vehicular networks can provide additional spectrum resources for vehicular communications; thus, they have the potential to be widely adopted in many scenarios, such as road transportation, railway transportation, aerospace, as well as military fields [3,4,5]. Vehicle detection and tracking is a critical technology that collects road traffic videos and exploits image processing approaches to guide traffic operations in cognitive radio-enabled vehicular networks [6]. Different from traditional techniques such as radar, lidar, RFID, or LASAR, vehicle detection and tracking has its own advantages in terms of real-time, low-cost, and high performance, and has shown effectiveness with a carefully designed detection and tracking model for intelligent traffic management [7]. However, the complex environment and changeable weather conditions pose an open problem for high-precision vehicle detection and tracking [8,9,10,11].

### 1.1. Literature Review

#### 1.1.1. Vehicle Detection

Many works have been devoted to investigating high-precision vehicle detection methods by now [12,13,14,15]. These works mainly focus on the two categories of vehicle detection technology based on traditional image processing technology or convolutional neural networks (CNN). The former category usually requires manual participation and guidance with poor robustness, while the latter one uses the CNN network to extract vehicle features, which is more robust and is suitable for many complex scenarios. In particular, the vehicle detection based on CNN can be further divided into two sub-categories: one-stage or two-stage vehicle detection methods [16]. One-stage vehicle detection methods, including RetinaNet [17], CFENet [18], CornerNet [19], YOLO [20], SSD [21], etc., can directly send the original image into the CNN model for vehicle feature extraction and obtain the position and category of the vehicle through regression and classification. Two-stage vehicle detection methods such as Cascade R-CNN [22], Faster R-CNN [23], Mask R-CNN [24], R-FCN [25], and SPP-Net [26] first extract the candidate regions of the vehicle in the image and then send them into the CNN model for feature extraction and classification.

Compared with the two-stage vehicle detection methods, the one-stage vehicle detection methods have the advantages of saving time and low cost. However, the vehicle classification accuracy of these methods is relatively low since they perform the object localization and classification tasks on an input image with a single neural network only one time. Moreover, it is notable that in real-time video analysis tools such as traffic monitoring, only Yolo can be used because of its high-speed detection. Based on the initial version of YOLO in [20], several algorithms including YOLOv2 [27], YOLOv3 [28], YOLOv4 [29], and YOLOv5 [30] were continuously developed to further improve the classification accuracy. Particularly, the open-source version of YOLOv5 can fully extract the features of the vehicle with high detection accuracy, and thus, has achieved a double harvest of speed and precision.

#### 1.1.2. Vehicle Tracking

With the help of vehicle models, the multi-target vehicle tracking technology based on CNN has also attracted much attention [31]. In [32], Bewley et al. selected the Faster R-CNN algorithm as the target detector and proposed a real-time tracking algorithm (SORT) to simultaneously track multiple targets based on the Kalman filter and the Hungarian matching algorithm. Unfortunately, the SORT algorithm only considers the motion characteristics of the target in the data association stage since the Kalman filter will have problems such as probability dispersion and data association failure when the targets are occluded. To address this problem, the authors in [33] considered both motion characteristics and appearance characteristics of the targets and proposed the improved DeepSORT algorithm, where the appearance features of the target through a CNN model are extracted after the detector detects the target. To further improve the complexity of the DeepSORT algorithm, Wang et al. [34] developed the JDE algorithm, which takes the target feature extraction network into the target detection network and directly outputs the target location and appearance features in the detection network. By using RetinaNet as the vehicle detector and embedding the instance-level vehicle feature extraction network in the detector model, Lu et al. [35] established the multi-target vehicle tracking model RetinaTrack to integrate vehicle motion characteristics and appearance characteristics for data association.

### 1.2. Contributions

It is worth noting that all methods in the aforementioned literature mainly focus on how to design a more efficient CNN network model to improve the detection speed of the target detection algorithm as much as possible. Although there exist several real-time vehicle detection and tracking methods, none of these approaches are sufficient to solve the vehicle detection and tracking problem of small target vehicles accurately. How to improve the detection and tracking accuracy of occluded small vehicles and reduce the number of vehicle identity switching instances has become an urgent problem [36]. As a step further towards the solution of the problem, this paper proposes a novel vehicle detection and tracking method for small target vehicles to achieve high detection and tracking accuracy based on the attention mechanism. Our main contributions are as follows:

- We develop a vehicle detection model YOLOv5-NAM based on the YOLOv5 model. By adding the spatial attention mechanism and channel attention mechanism module into the model, the detection accuracy of small vehicles is improved. An SD-NMS method based on the idea of the penalty function is further proposed to solve the problem of missed detection of vehicles in dense scenes.
- We propose a real-time multi-vehicle tracking method JDE-YN based on the JDE algorithm, which improves the tracking accuracy of the vehicles and reduces the number of vehicle identity switching instances. Based on direction correction, we also develop a cascade matching algorithm to solve the problem of vehicle identity switching caused by occlusion.

The rest of this paper is organized as follows. We introduce the system model of detection-based vehicle tracking in Section 2 and provide a detailed description of our vehicle detection and tracking method in Section 3. Section 4 presents the experiments and analysis of results, and Section 5 concludes the paper.

## 2. System Model

**Figure 1:**
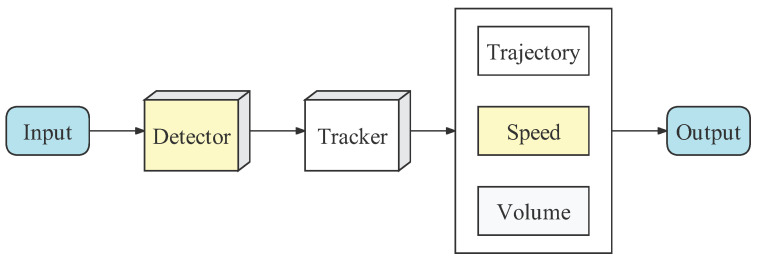
The detection-based vehicle tracking model.

As shown in Figure 1, we consider a vehicle detection and tracking model, which consists of a detector and a tracker. The detector is responsible for detecting vehicles in each frame, while the tracker aims to correlate vehicles in adjacent frames to form a complete vehicle trajectory.

### 2.1. Detection Model

**Figure 2:**
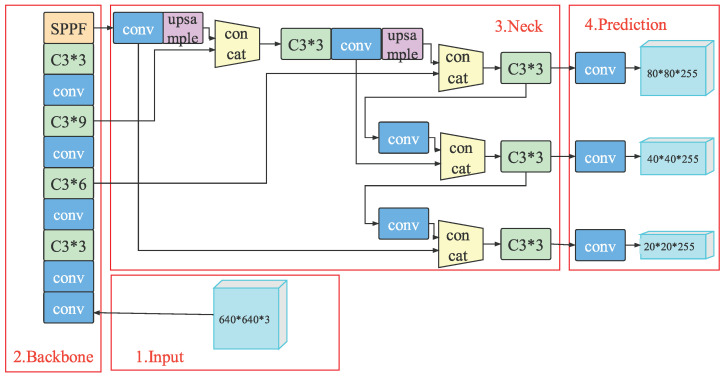
The YOLOv5 network model.

Recently, the YOLO detection model in [20] was iterated from YOLOv1 to YOLOv5, which integrates the existing advanced research results and improves the detection accuracy and speed of the model. For a better understanding of our vehicle detection model, we first introduce the 6.0 version of the YOLOv5s network model, as shown in Figure 2. Here, based on different functions, the whole model can be divided into four parts: Input, Backbone, Neck, and Prediction Head.

#### 2.1.1. Input

The Input part provides data for model training and performing preprocessing operations on the experimental dataset. The YOLOv5 algorithm mainly uses Mosaic data enhancement and adaptive anchor box strategy on the Input to preprocess the original dataset. Mosaic data enhancement is borrowed from CutMix data enhancement [37]. The adaptive anchor box strategy solves the problem of differences in the aspect ratio of different targets. For example, the aspect ratio of the anchor box of the pedestrian is often greater than 1, while the aspect ratio of the anchor box of vehicles is just the opposite. The YOLOv5 algorithm introduces an adaptive anchor box strategy at the Input model that can automatically modify the preset anchor box during training.

#### 2.1.2. Backbone

**Figure 3:**
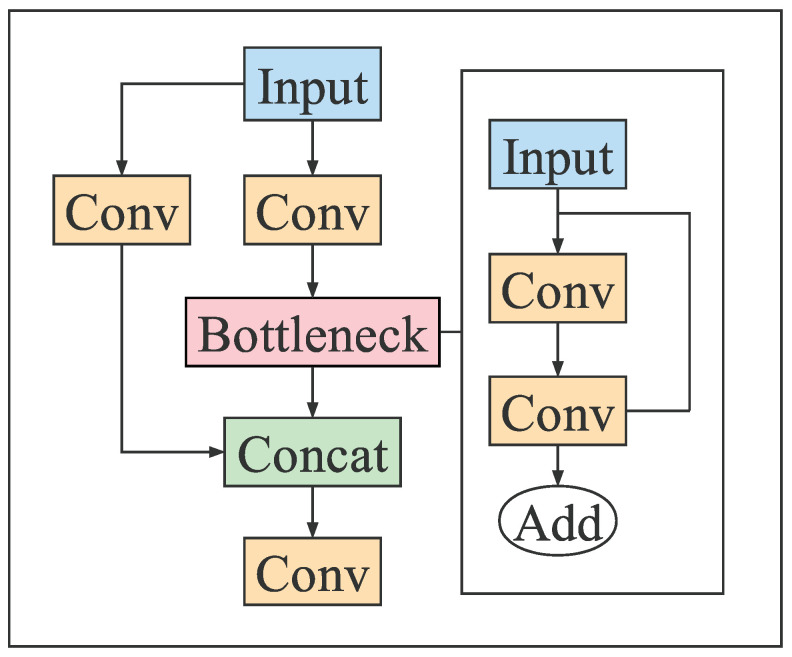
C3 structure.

**Figure 4:**
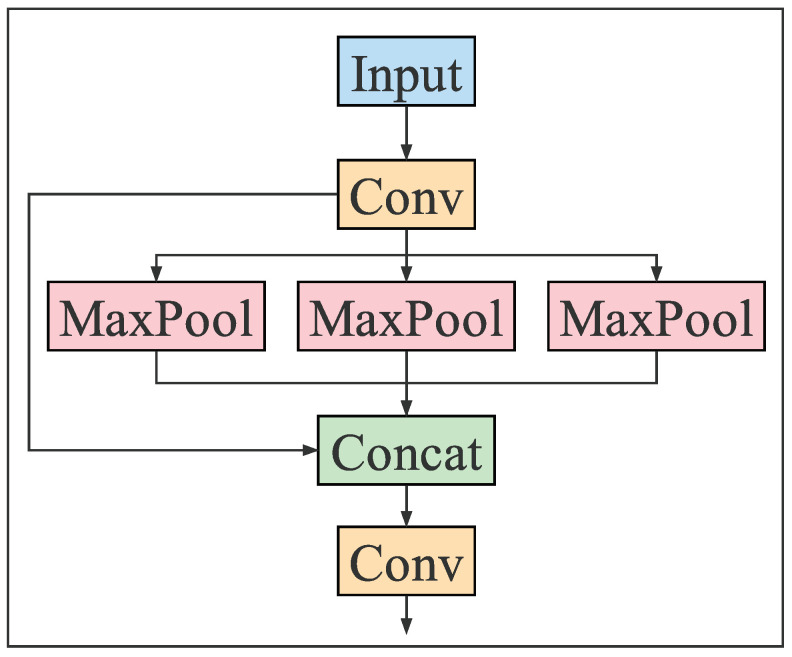
SPPF structure.

The Backbone is an indispensable part of the vehicle detection network model, which extracts the features of the original image through different CNN operations for subsequent classification and regression operations. After several years of development, researchers have designed some excellent Backbones, such as VGG, Darknet53, ResNet, and MobileNet. The Backbone of YOLO series algorithms is based on Darknet53 for extended research. The latest YOLOv5 algorithm Backbone consists of Conv, C3, and SPPF modules. The structures of C3 and SPPF are shown in Figure 3 and Figure 4, respectively.

#### 2.1.3. Neck

**Figure 5:**
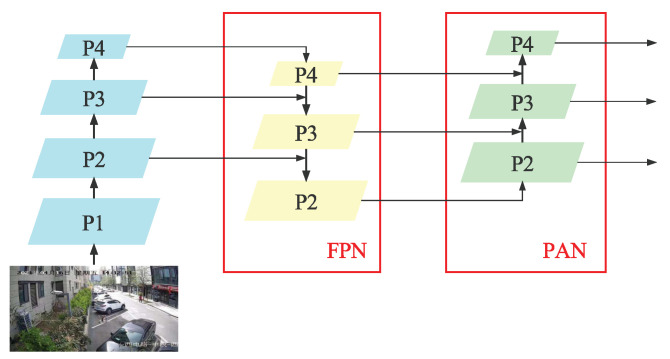
The FPN+PAN structure.

The Neck is between the Backbone and the Prediction Head, mainly to fuse the image features extracted by the Backbone. The YOLOv5 algorithm selects the FPN+PAN network structure to fuse image features in the Neck, as shown in Figure 5. The FPN network is responsible for passing the high-level semantic information down and merging with the Backbone. The PAN structure is responsible for the low-level feature information upward and merging with the FPN network. The final output feature map is the input feature map of the Prediction Head for target classification and prediction.

#### 2.1.4. Prediction Head

The Prediction Head is the most important part of the vehicle detection network model. Its function is to expand the feature map into a one-dimensional vector and perform vehicle detection and classification operations on this one-dimensional vector. The YOLOv5 algorithm has three Prediction Heads to detect large targets, medium targets, and small targets. The Prediction Head of 20×20×255 has the largest receptive field and is used to predict large targets. The Prediction Head of 40×40×255 is used to predict medium targets. The Prediction Head of 80×80×255 has the smallest receptive field and is used to predict small targets. The loss function will continually correct the accuracy of classification and regression, and the latest YOLOv5 algorithm chooses GIoU_Loss for training. There are many redundant target boxes in the output result of the Prediction Head, and the non-maximum suppression operation is used to screen out the detection box with high confidence as the final detection result of the target.

### 2.2. Tracking Model

The Kalman filter (KF) is an optimization method to solve the optimal state estimation of the system [38]. It usually serves as the tracker model for vehicle tracking tasks. By constructing the state transition equation and observation equation of the system, the KF algorithm continuously observes and estimates the state of the system, so that the observed value and the estimated value are as close to the real value as possible, to obtain the optimal estimation of the target motion. The general expressions of the state transition equation and observation equation of the KF algorithm are defined as follows:

(1) $$\begin{array}{cc} x_{t} & =Ax_{t-1}+Eu_{t-1}+q_{t}, \end{array}$$

(2) $$\begin{array}{cc} y_{t} & =Hx_{t}+r_{t}, \end{array}$$

where $x_{t}$ and $y_{t}$ are the system state real value and observed value at time *t*, respectively; *A* is the system state transition matrix; *H* is the system state observation matrix; *E* and $u_{t-1}$ are the system model parameters; and $q_{t}$ and $r_{t}$ are the system state transition noise and observation noise, respectively, which are subject to Gaussian distribution. The realization process of the algorithm mainly includes three steps: prediction, filter estimation, and parameter update. The specific process is as follows:

(1) Calculate the system state estimated value ${x_{t}}^{\prime }$ through the system state transition matrix *A*, and calculate its covariance matrix ${\Omega_{t}}^{\prime }$ with the system state real value:

(3) $$\begin{array}{cc} {x_{t}}^{\prime } & =Ax_{t-1}+Eu_{t-1}, \end{array}$$

(4) $$\begin{array}{cc} {\Omega_{t}}^{\prime } & =A\Omega_{t-1}A^{T}+Q, \end{array}$$

where *Q* is the covariance matrix of the system state transition noise $q_{t}$.

(2) Calculate the KF gain ${K_{t}}^{\prime }$ of the system at time *t* through the system state observe matrix *H* and the covariance matrix ${\Omega_{t}}^{\prime }$ obtained in (4), and use ${K_{t}}^{\prime }$ to estimate the system state at this time:

(5) $$\begin{array}{cc} {K_{t}}^{\prime } & ={\Omega_{t}}^{\prime }H^{T}{\left(H{\Omega_{t}}^{\prime }H^{T}+R\right)}^{-1}, \end{array}$$

(6) $$\begin{array}{cc} x_{t} & ={x_{t}}^{\prime }+{K_{t}}^{\prime }\left(y_{t}-H{x_{t}}^{\prime }\right), \end{array}$$

where *R* is the covariance matrix of the system state observation noise $r_{t}$.

(3) Update the covariance matrix $\Omega_{t}$ of the system state estimated value and the system state real value to prepare for the next recursion:

(7) $$\Omega_{t}=\left(1-{K_{t}}^{\prime }\right){\Omega_{t}}^{\prime }.$$

By setting the initial system state estimation value $x_{t}$ and the system state observation value $y_{t}$, and repeating the above algorithm process, we can obtain the latest values of $x_{t}$ and $y_{t}$, and obtain the optimal estimation of the system state continuously. In the vehicle tracking tasks, the motion state estimation model of the vehicle is established based on the KF algorithm to correct the vehicle position, which makes up for the deficiency of the vehicle detector and improves the tracking accuracy of the vehicle tracking algorithms.

## 3. Vehicle Detection and Tracking Method

By integrating the NAM attention module into the YOLOv5s network model, we build a high-precision, real-time vehicle detection model YOLOv5-NAM and optimize its loss function and NMS method to improve the detection accuracy. Then, we propose a high-precision, real-time vehicle tracking method JDE-YN, where the appearance feature extraction network of the vehicle is embedded in the detection head of the YOLOv5-NAM model.

### 3.1. The YOLOv5-NAM Vehicle Detection Model

As an extension of the CBAM attention module, NAM is a lightweight and efficient new attention module achieved by redesigning the channel and spatial attention submodule based on normalization technology [39]. While the past research work in this field has focused on improving the image of the significant characteristics and ignores the problem of the striking feature of the image, the NAM attention module chooses the weight of the image channel and spatial information as a measure of image characteristics of significance, through a normalized scaling factor to represent the importance of the weight, and suppress the unimportant channel information and pixel information in the image.

**Figure 6:**
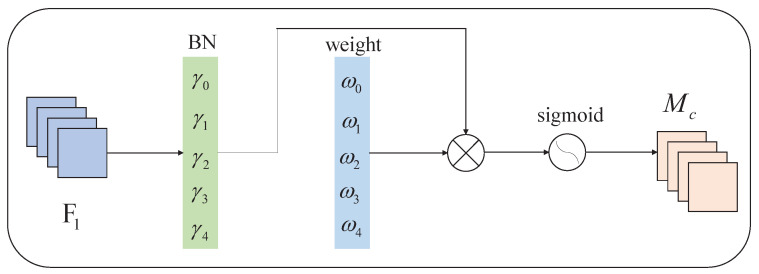
Channel attention submodule of NAM.

Figure 6 shows the structure of the NAM channel attention submodule. The channel attention module is mainly for the model to acquire the ability of “what to see”, corresponding to the classification problem of the vehicle target detection task. Given the input feature map $F_{1}$, the module first calculates the scale factor $\gamma_{0},\gamma_{1},\ldots ,\gamma_{n}$ of each channel of $F_{1}$ through the regular normalization operation; then, it calculates the weight value of the scale factor of each channel of $F_{1}$ as follows:

(8) $$\omega_{i}=\frac{\gamma_{i}}{\sum_{j=0}\gamma_{j}}.$$

The weight value of each channel is applied to the original feature map $F_{1}$ as a penalty term, and the final image channel weight coefficient $M_{c}$ is obtained through the sigmoid activation function.

**Figure 7:**
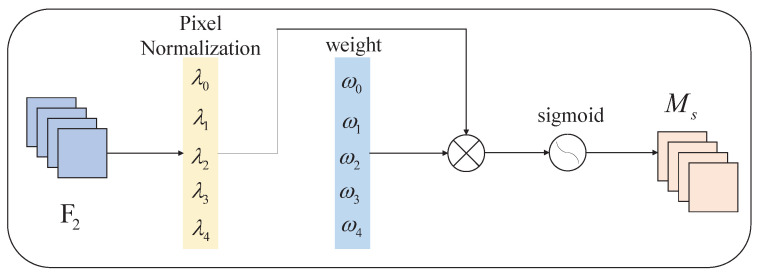
Spatial attention submodule of NAM.

Figure 7 shows the structure of the NAM spatial attention submodule. The spatial attention module is mainly for the model to acquire the ability of “where to look”, corresponding to the positioning problem in the vehicle detection task. Given the input feature map $F_{2}$, the module first calculates the scale factor $\lambda_{0},\lambda_{1},\ldots ,\lambda_{n}$ of each pixel in $F_{2}$ through normalization operation; then, it calculates the weight value of the scale factor of each pixel in $F_{2}$ as follows:

(9) $$\omega_{i}=\frac{\lambda_{i}}{\sum_{j=0}\lambda_{j}}.$$

The weight value of each pixel is applied to the original feature map $F_{2}$ as a penalty term, and the final image channel weight coefficient $M_{s}$ is obtained through the sigmoid activation function.

**Figure 8:**
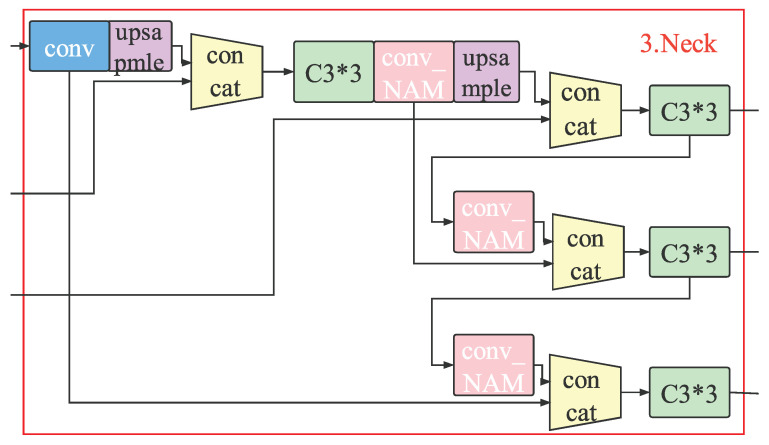
The Neck of the YOLOv5-NAM network model.

As shown in Figure 8, we add the NAM attention module to the Neck of the YOLOv5s network model to build a YOLOV5-NAM vehicle detection model, where we aim to improve the detection ability for small vehicles, increase the vehicle detection accuracy, and reduce the rate of missed detection and error detection. Note that we adopt the mosaic data enhancement strategy at the Input for image preprocessing. Here, the size of the input image is 640*640*3 and the Backbone network is consistent with the original YOLOv5s model. The Neck still selects the FPN+PAN structure but the difference is that the convolution module before the last three Concat connection modules is replaced with the conv_NAM module to make the model pay attention to more features of the vehicles so as to improve the overall detection accuracy of the model.

### 3.2. The Loss Function

The loss function of the YOLOv5 model training mainly includes three parts: the target classification loss, the target bounding box loss, and the target confidence loss. Formally, the YOLOv5 loss function is given by

(10) $$L_{YOLOv5}=L_{lcls}+L_{lobj}+L_{lbox},$$

where the target classification loss and the target confidence loss used the cross-entropy loss function for training, and the target bounding box loss used the Generalized Intersection over Union (GIoU) loss function for training.

The calculation process of cross-entropy loss of target classification loss in the YOLOv5s model is as follows:

(11) $$\begin{array}{cc} L_{lcls} & =\sum_{i=0}^{S^{2}}\sum_{j=0}^{B}1_{i,j}^{obj}\sum_{c\in classes}[y_{i}\left(c\right)log\left(p_{i}\left(c\right)\right)\\ \; & +\left(1-y_{i}\left(c\right)\right)log\left(1-p_{i}\left(c\right)\right)]. \end{array}$$

The calculation process of cross-entropy loss of target confidence loss in the YOLOv5s model is as follows:

(12) $$\begin{array}{cc} L_{lobj} & =\sum_{i=0}^{S^{2}}\sum_{j=0}^{B}1_{i,j}^{noobj}\left[\left(y_{i}-1\right)log\left(1-p_{i}\right)-y_{i}log\left(p_{i}\right)\right]\\ \; & +\sum_{i=0}^{S^{2}}\sum_{j=0}^{B}1_{i,j}^{obj}\left[y_{i}log\left(p_{i}\right)+\left(1-y_{i}\right)log\left(1-p_{i}\right)\right]. \end{array}$$

Compared with the original loss function of the YOLOv5s model, we choose the CIoU_Loss as the target bounding box’s loss function for model training. The GIoU_Loss only considers the overlap area between the target bounding boxes and ignores the influence of other influencing factors on model training. Based on the GIoU_Loss, the CIoU_Loss [40] adds the center point distance and the aspect ratio between the target bounding boxes. The calculation formula is as follows:

(13) $$L_{CIoU}=1-\frac{d^{2}\left(P,G\right)}{c^{2}}-\alpha v.$$

The complete calculation process of the loss function for the YOLOv5-NAM model training is as follows:

(14) $$\begin{array}{cc} L & =L_{CIoU}\\ \; & +\sum_{i=0}^{S^{2}}\sum_{j=0}^{B}1_{i,j}^{noobj}\left[\left(y_{i}-1\right)log\left(1-p_{i}\right)-y_{i}log\left(p_{i}\right)\right]\\ \; & +\sum_{i=0}^{S^{2}}\sum_{j=0}^{B}1_{i,j}^{obj}\left[y_{i}log\left(p_{i}\right)+\left(1-y_{i}\right)log\left(1-p_{i}\right)\right]\\ \; & +\sum_{i=0}^{S^{2}}\sum_{j=0}^{B}1_{i,j}^{obj}\sum_{c\in classes}[y_{i}\left(c\right)log\left(p_{i}\left(c\right)\right)\\ \; & +\left(1-y_{i}\left(c\right)\right)log\left(1-p_{i}\left(c\right)\right)]. \end{array}$$

### 3.3. The SD-NMS Method

The Non-Maximum Suppression (NMS) algorithm is a post-processing method for vehicle detection tasks. To increase the detection accuracy and reduce the missed detection rate in the anchor-based vehicle detection model, researchers usually generate many candidate boxes in the initial stage and then match different scores for different candidate boxes in the analysis stage, which results in a large number of redundant boxes. How to select the correct prediction boxes from the candidate boxes as the output boxes is a key step to determine the detection accuracy of the vehicle detection model.

Algorithm 1 is the pseudocode of the NMS algorithm, where B is a candidate set of the boxes position, S is a candidate set of the boxes socre, $N_{t}$ is a screening threshold, and D is a set of the prediction boxes. When set B is not null, select the maximum score m from set S; add the corresponding candidate box $b_{m}$ in D into temporary set M; and move elements from set B to set D, which are in set M. Exclude the candidate box whose IoU value with $b_{m}$ is greater than $N_{t}$ in set B and set S. Loop iteration until set B is empty; the final output D and S is the income forecast box position and the corresponding score.
**Algorithm 1:** The Non-Maximum Suppression algorithm1 While B≠empty2 m←argmaxS3 $M\leftarrow b_{m}$4 D←D∪M;B←B−M5 For $b_{i}\in B$6 If $iou\left(M,b_{i}\right)$7 $B\leftarrow B-b_{i};S\leftarrow S-s_{i}$

**Figure 9:**
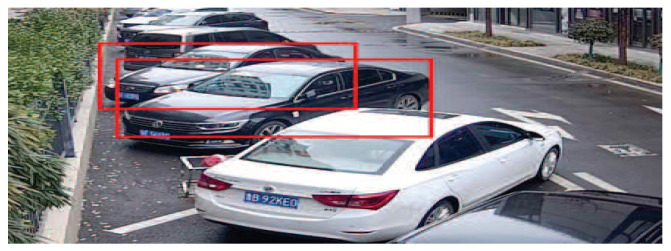
Vehicle overlapping.

The NMS algorithm can obtain better suppression effects in single-target and sparse multi-target scenes. However, the distance between the two targets is close in the dense multi-target case, and there is often a big overlap area between the real targets, as shown in Figure 9. If the NMS algorithm is directly applied, the detection boxes of the vehicle may be deleted by mistake, resulting in missed detection.

To solve this problem, we further propose a Soft-DIoU NMS (SD-NMS) method based on DIoU-NMS [40]. The overall process of this method is consistent with Algorithm 1; we choose the DIoU distance as the new distance metric. The first step is to select the maximum score of the prediction boxes from the candidate boxes set S. The difference is that the IoU-NMS method directly deleted the candidate boxes with an IoU value over $N_{t}$. We will give a penalty item based on the initial score for the candidate boxes whose DIoU value is greater than $N_{t}$, and the penalty intensity is determined by the DIoU value. The calculation process is as follows:

(15) $$\begin{array}{c} \hat{S_{i}}=\left\{\begin{array}{cc} S_{i} & DIoU\left(B,b_{i}\right)<N_{t},\\ S_{i}\left(1-|DIoU\left(B,b_{i}\right)|\right) & DIoU\left(B,b_{i}\right)\geq N_{t}. \end{array}\right. \end{array}$$

We can obtain a group of new candidate boxes by continuously punishing candidate boxes and then deleting the low confidence prediction boxes by a uniform confidence threshold, which increases the detection accuracy and reduces the missed detections.

### 3.4. The Vehicle State Estimation Model

We reconstruct the vehicle state estimation model by introducing process noise into the system. The motion state of the vehicle is expressed as an 8-dimensional vector:

(16) $$S={\left[x,y,h,r,v_{x},v_{y},v_{h},v_{r}\right]}^{T},$$

where *x* and *y* are the horizontal and vertical coordinates of the vehicle center point in the image; *h* is the height of the vehicle boxes; *r* is the aspect ratio of the vehicle boxes; and $v_{x}$, $v_{y}$, $v_{h}$, and $v_{r}$ are the speed components in four directions, respectively.

Suppose $S_{t}$ is the position of the target vehicle at time *t*, $v_{t}$ is the speed of the target vehicle at time *t*, and the acceleration of the target vehicle is *a*; then, the position of the target vehicle and the speed of the target vehicle at time t+1 is given by

(17) $$\begin{array}{cc} S_{t+1} & =S_{t}+v_{t}\Delta t+\frac{1}{2}a{\Delta t}^{2}, \end{array}$$

(18) $$\begin{array}{cc} v_{t+1} & =v_{t}+a\Delta t, \end{array}$$

where Δt is the interval from *t* to t+1.

Therefore, we can build the vehicle state prediction model as follows:

(19) $$S_{t+1}=AS_{t}+Ea,$$

where *A* is the vehicle state transition matrix and *E* is the noise control matrix in the process of vehicle driving. Combining with the 8-dimensional vehicle state features we established in (16), it is easy to obtain the following:

(20) $$A_{t}=\left[\begin{array}{cccccccc} 1 & 0 & 0 & 0 & \Delta t & 0 & 0 & 0\\ 0 & 1 & 0 & 0 & 0 & \Delta t & 0 & 0\\ 0 & 0 & 1 & 0 & 0 & 0 & \Delta t & 0\\ 0 & 0 & 0 & 1 & 0 & 0 & 0 & \Delta t\\ 0 & 0 & 0 & 0 & 1 & 0 & 0 & 0\\ 0 & 0 & 0 & 0 & 0 & 1 & 0 & 0\\ 0 & 0 & 0 & 0 & 0 & 0 & 1 & 0\\ 0 & 0 & 0 & 0 & 0 & 0 & 0 & 1 \end{array}\right],$$

(21) $$\begin{array}{c} E_{t}={\left[\frac{1}{2}{\Delta t}^{2}\;\frac{1}{2}{\Delta t}^{2}\;\frac{1}{2}{\Delta t}^{2}\;\frac{1}{2}{\Delta t}^{2}\;\Delta t\;\Delta t\;\Delta t\;\Delta t\right]}^{T}. \end{array}$$

Here, we can build the vehicle state observation model as follows:

(22) $$P_{t}=HS_{t},$$

where *H* is the system state observation matrix. Consider that we can only observe the vehicle position in the image through the video and the vehicle speed cannot be directly obtained. The observation matrix *H* is as follows:

(23) $$H=\left[\begin{array}{cccccccc} 1 & 0 & 0 & 0 & 0 & 0 & 0 & 0\\ 0 & 1 & 0 & 0 & 0 & 0 & 0 & 0\\ 0 & 0 & 1 & 0 & 0 & 0 & 0 & 0\\ 0 & 0 & 0 & 1 & 0 & 0 & 0 & 0 \end{array}\right],$$

In view that vehicle tracking in road traffic scenarios is susceptible to adverse weather and other external factors, we introduce system noise and observation noise to modify the model, and the complete vehicle state estimation model expression is as follows:

(24) $$\begin{array}{cc} S_{t+1} & =A_{t}S_{t}+C_{t}a+Q, \end{array}$$

(25) $$\begin{array}{cc} P_{t} & =HS_{t}+R. \end{array}$$

Based on the above analysis, we can use the YOLOv5-NAM vehicle detector to achieve multi-target vehicle tracking.

### 3.5. The Cascade Matching Algorithm Based on Direction Correction

The traditional multi-target tracking algorithm usually chooses the Hungarian matching algorithm as the data association strategy of the target between adjacent frames. The algorithm cost matrix is built based on the target motion characteristics and appearance characteristics. The distance measurement method of the target motion feature is the Mahalanobis distance between the output result of the state estimation model and the output result of the detector. Mahalanobis distance weakens the correlation between variables and makes the distance measurement of the target more accurate. The distance measurement method of target appearance characteristics is the cosine distance between the output result of the state estimation model and the output of the feature extraction network. Cosine distance depicts the appearance of the similarity between the target and makes up for the deficiency of the distance measurement method of target motion characteristics when the target motion is uncertain.

**Figure 10:**
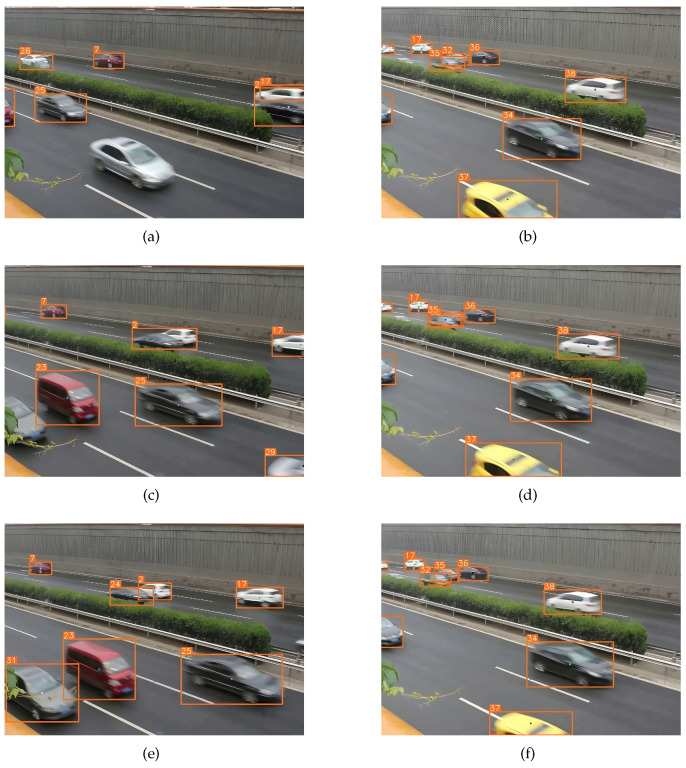
Data association failure. (**a**) frame 115; (**b**) frame 187; (**c**) frame 132; (**d**) frame 188; (**e**) frame 137; (**f**) frame 190.

The inter-class similarity and the intra-class difference between the vehicles will lead to the failure of vehicle data association and also vehicle identity switching when the vehicles occlude each other during the driving process. In Figure 10, track 2 and track 17, respectively, belong to the black car and the white car in frame 115. The black car occluded the white car in frame 132, resulting in the white car miss detection. At the time, a white vehicle appeared and was mistakenly associated with track 17. The occlusion ended in frame 137 but the algorithm had assigned track 17 to the white vehicle; so, the white car had to be reassigned to another, causing the vehicle identity to switch. In panels (b), (d), and (f), track 32 and track 35 belong to the car and taxi in frame 187. The car and taxi occluded each other in frame 188. The occlusion ended in frame 190 but the data association algorithm assigned track 32 to the taxi and track 35 to the car, causing the vehicle identity to switch between the taxi and the car.

**Figure 11:**
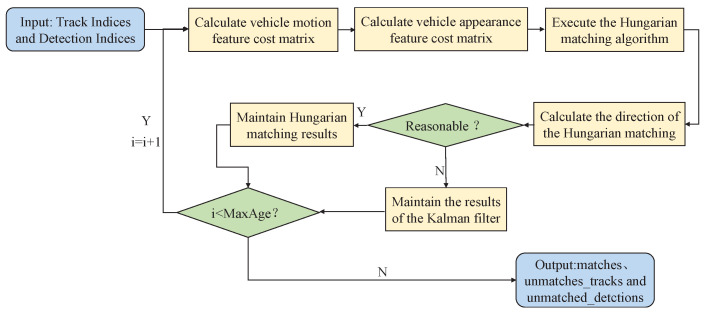
The cascade matching algorithm based on direction correction.

It is not difficult to find that when the vehicle identity switches due to occlusion, there is a “jump” phenomenon in the vehicle trajectory. In other words, the vehicle undergoes an abnormal angular rotation, which is almost impossible to occur in the driving environment. Therefore, we propose a cascade matching algorithm based on direction correction. Whether the result of data association is reasonable or not is judged by integrating the directional features of vehicles. If reasonably performed, the data association result is confirmed; otherwise, we predict that maybe occlusion occurred, cancel the data association result, and maintain Kalman filter prediction results, as shown in Figure 11.

## 4. Detection and Tracking Experience Results

### 4.1. Detection Results on UA-DETRAC

The UA-DETRAC dataset is a large, open-source dataset for multi-vehicle detection and tracking [41]. The dataset was taken from the video surveillance of 24 real roads in Beijing and Tianjin, covering four different weather conditions: sunny, rainy, cloudy, and night. The researcher divided the vehicles into four types: cars, buses, vans, and other vehicles. On this basis, they marked 8250 vehicles and 1.21 million labels in total.

Since the UA-DETRAC dataset comes from multiple videos, the vehicle difference in most adjacent video frames is not evident. Direct application in the training of the YOLOv5-NAM model will lead to data redundancy. Therefore, we extract the original video every five frames, which can reduce the sample size of the dataset and reduce the training time of the model. At the same time, this method can also avoid the over-fitting phenomenon caused by repeated learning of the same feature. We select 16,542 images and 102,562 vehicle labels from the original UA-DETRAC train set as the new train set and select 11,235 images and 86,559 vehicle labels from the original UA-DETRAC test set as the new test set. Based on the above vehicle dataset, we set the initial learning rate as 0.01, the batch number as 32, and the epoch as 100. Here, we set a relatively high confidence level to compare the visual experiment results.

Table 1 shows the influence of network structure improves on model performance. As can be seen from the table, after adding the NAM attention module, the number of network layers in this model increases from 213 to 219 layers. The model size increases from 13.7 M to 13.9 M. The FPS of the model slightly decreases by 0.4. However, the overall mAP value of the model increases by 0.9%, indicating that the YOLOv5-NAM vehicle detection model is effective and the detection accuracy of the model increases with the sacrifice of a small amount of space and speed.

Table 2 shows the influence of the loss function on improving the model performance. We can see from the table that the loss function does not directly influence the model layers, the model size, and the FPS indicators. The reason is that the loss function acts on the model training stage, while the number of model layers and model size are determined before the model training. The FPS indicator is the product of the model testing stage; so, it is not affected by the loss function. The direct influencing factor of the loss function is the value of mAP. When we use the CIoU_Loss to replace the GIoU_Loss, the YOLOv5s and YOlOv5-NAM models’ mAP values increase by 0.2% and 0.3%, respectively. This shows that replacing the loss function training model can improve the detection accuracy of the model.

Table 3 shows the influence of the NMS method on model performance. As can be seen from the table, the model layers and model size are not affected by the NMS method, which verifies that the non-maxima processing is a post-processing method and acts on the model testing stage. We can also find that the FPS index of the model decreases slightly after replacing the NMS method of the YOLOv5s and YOLOv5-NAM models with the SD-NMS method; the FPS indexes of the YOLOv5s and YOLOv5-NAM models decrease by 3 and 2.8, respectively. It is mainly because the SD-NMS method adopts the idea of cyclic suppression, which requires a longer processing time than the NMS method. However, after replacing the NMS method with the SD-NMS method, the mAP of the YOLOv5s and YOLOv5-NAM models increases by 0.2%. It is worth mentioning that when the targets are denser, the improvement effect will be even higher.

To further analyze the influence of the method on the detection accuracy of the four vehicle categories—cars, buses, vans, and other types of vehicles—we also analyze the detection accuracy of the YOLOv5s and YOLOv5-NAM model, and the NMS method and SD-NMS method, for single-vehicle targets. The experimental results are shown in Table 4, where the evaluation indicators are the AP value of a single category and the mAP of all categories. According to the Table, after adding the NAM attention mechanism into the original YOLOv5s model, the overall mAP value of the model increases by 1.4%. The AP value of cars increases by 0.3%, the AP value of buses increases by 2.7%, the AP value of vans slightly decreases, and the AP value of other vehicles increases by 3.6%. When the detection results of the YOLOv5s algorithm are post-processed by the SD-NMS method, the overall mAP value of the model does not change significantly, only increasing by 0.1%, while the AP value of different types of vehicles varies. The AP value of cars increases by 1.5%, the AP value of others increases by 0.4%, and the AP value of buses and vans slightly decrease. When the NAM attention mechanism and the SD-NMS method are added to the YOLOv5s algorithm, the overall mAP value of the model increases by 1.6%, among which the AP of cars increases by 2.3%, the AP of buses increases by 1.3%, the AP of other vehicles increases by 4.7%, and the AP of vans has a slight decrease.

The experimental results show that the NAM attentional mechanism module has a significant effect on improving the overall mAP value of the model. No matter the NAM attention mechanism or the SD-NMS method, the AP values of other vehicles of a single class are improved to varying degrees, mainly because other vehicles in the UA-DETRAC dataset are mostly marked instances of vehicles with a small volume or serious occlusion, which is consistent with the fact that our proposed method can improve the detection accuracy of vehicles with small targets; the van is mostly a large target, so the accuracy of our proposed method is slightly lost. This suggests that our proposed method has a certain effect on improving the overall detection accuracy of vehicles and a significant effect on improving the detection accuracy of small target vehicles.

**Figure 12:**
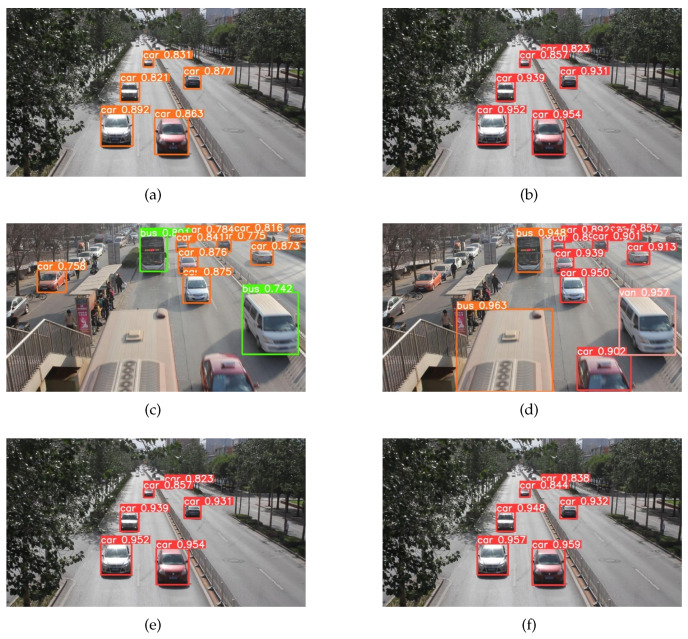
The visual detection results of the YOLOv5s and the YOLOv5-NAM models.

The visualization results of the YOLOv5s model and the YOLOv5-NAM vehicle detection model are shown in Figure 12, where panels (a) and (c) are the detection results of the YOLOv5s model training on the COCO dataset and panels (b) and (d) are the detection results of the YOLOv5-NAM vehicle detection model training on the UA-DETRAC dataset. It is not difficult to find that after retraining on the UA-DETRAC dataset, the YOLOv5-NAM vehicle target detection model greatly improves the detection accuracy of vehicles. The detection accuracy of a single-vehicle target is above 0.9 on average, which greatly improves the detection accuracy of vehicles with small targets. The new model also improves the error detection of the YOLOv5s model to some extent. For example, the YOLOv5s algorithm mistakenly detects the van as a bus in subgraph (c) but the YOLOv5-NAM algorithm correctly detects the van in subgraph (d).

To eliminate the influence of the dataset, we retrain the YOLOv5s model on the UA-DETRAC dataset. Panels (e) and (f) in Figure 12 are the detection results of the YOLOv5s and YOLOV5-NAM model after retraining on the UA-DETRAC dataset, respectively. As can be seen from the figure, the vehicle detection accuracy of the YOLOv5-NAM model is improved to varying degrees compared with the YOLOv5s model, especially for small target vehicles, which confirms that the detection effect of our proposed method is indeed improved for small target vehicles.

### 4.2. Detection Results on COCO

To verify that the YOLOv5-NAM model also has a certain reference significance for general object detection tasks, we retrain the YOLOv5-NAM model on the COCO2017 dataset. The training results are compared with the training results of mainstream target detection algorithms on this dataset. The evaluation index is the AP value officially provided by COCO. The results are shown in Table 5, where $AP_{50}$ and $AP_{75}$ are the AP values when the IoU values are 0.5 and 0.75, respectively; $AP_{S}$, $AP_{M}$, and $AP_{L}$ are the AP value of small targets, medium targets, and big targets, respectively.

From Table 5, the AP value of our proposed method is higher than some classical object detection algorithms such as Faster R-CNN, SSD, and YOLOv3, and slightly lower than the RetainNet and CenterNet algorithms. Our research is mainly oriented to road traffic scenarios and has high requirements for the real-time performance of the algorithm; therefore, we choose the lightest YOLOv5s model for improvement. However, the YOLOv5x model with a higher AP value is not chosen for improvement, which is also the main reason why the AP value of our proposed method is slightly lower. If the improved idea of the paper is applied to the YOLOv5x model, the AP value will be greatly improved but the reasoning speed of the model will be greatly reduced, which does not apply to our scenario. Although the AP value of our proposed method is lower than the CenterNet and RetinaNet algorithms, the detection accuracy of the small target $AP_{S}$ is higher than the CenterNet and the gap between our proposed method and the RetinaNet is also significantly reduced, which indicates that our proposed method has a good effect on improving the detection accuracy of small targets.

### 4.3. Tracking Results on UA-DETRAC

We choose the UA-DETRAC dataset for model training, choose 60 video sequences as the training set for model training, and choose 40 video sequences as the test set to measure the training results of the model. Every video sequence is split into a single frame and encapsulated in the same folder, and all image annotation information under each folder is encapsulated in an xml file. The xml file is listed in the unit of video frames and describes in detail the vehicle ID, vehicle position coordinates, vehicle speed, vehicle rotation angle, and vehicle type in each frame. In order to facilitate the training of the YOLOv5-NAM vehicle target detection model, we transform the xml file annotation format into VOC annotation format through the custom tools, retain the vehicle ID and location coordinate information, and set the vehicle type to car uniformly.

**Figure 13:**
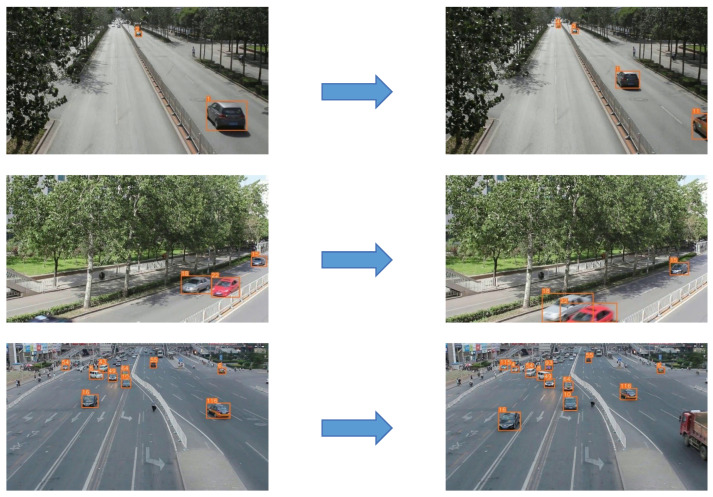
The vehicle target tracking results.

We use kmeans algorithm to obtain a set of optimal anchor boxes on UA-DETRAC dataset in advance, which is $[\left(25,20\right),\left(33,28\right),\left(44,34\right),\left(53,47\right),\left(68,38\right)$, $\left(178,213\right),\left(96,49\right),$$\left(131,62\right),\left(97,89\right),\left(159,104\right)$, $\left(255,134\right),\left(72,61\right)]$. During the experiment, the weight of the YOLOv5s pre-training model was used for training, and the initial learning rate was set to 0.01, the training loss of the model was optimized by Adam function, the batch processing size was set to 32, and the image size was set to 960*540. The weight of the trained model is tested on the test set for vehicle target tracking, and some of the target vehicle tracking results are shown in Figure 13.

To verify the effectiveness of the proposed multi-target vehicle tracking method, we also carry out comparative experiments with benchmark methods such as SORT, DeepSORT, and JDE. The experimental dataset was the UA-DETRAC vehicle tracking dataset, and the experimental parameters remained unchanged. The evaluation indexes of experimental results are MOTA, MOTP, MT, ML, IDs, and the real-time processing speed FPS of the algorithm; the results are shown in Table 6.

From Table 6, the MOTA and MOTP values of the four methods in the table are low on the UA-DETRAC dataset, maintaining between 0.3 and 0.4, which is mainly because the UA-DETRAC dataset covers a variety of weather environments and vehicle targets of different scales, which challenge the performance of the vehicle target detector, resulting in low MOTA and MOTP values. However, our proposed method still achieves the highest MOTA value of 0.343, and the MOTP value is only 0.1 percentage points lower than the highest JDE algorithm. Observation of the IDs column is not hard to find, and our proposed method obtained the identity of the switching times at least; this is mainly because our method integrates the directional features of vehicles in the data association stage, which reduces the error rate of data correlation, thereby reducing the switching times, and proving the effectiveness of our data correlation strategy. By observing the FPS column, it can be found that the SORT algorithm has the highest FPS but its IDs are too high to be of practical application value. The DeepSORT algorithm can significantly reduce IDs by integrating vehicle appearance features for data association; however, its FPS value also drops sharply, which cannot meet the requirements of real-time applications. The JDE method embedded the vehicle appearance feature extraction network into the detection network, which greatly improved the FPS value of the algorithm. Our proposed method is further improved based on the JDE algorithm, under the premise of losing a small part of FPS; the IDs of vehicle targets are further reduced by 15% compared with the JDE algorithm, which can support the application requirements of vehicle target tracking with high accuracy and high real-time performance.

## 5. Conclusions

This paper studied the small target vehicle detection and occlusion vehicle tracking. Based on the classical YOLOv5s model, we developed a new vehicle detection model (YOLOv5-NAM). By exploiting the YOLOv5-NAM model as the vehicle detector, we also proposed a real-time small target vehicle tracking method (JDE-YN). Our experiment results on the UA-DETRAC and COCO datasets demonstrated that the mAP value increased by 1.6% compared with the YOLOv5s model, the MOTA value of vehicle tracking increased by 0.9% compared with the JDE algorithm, and the identity switching times of vehicles decreased by 15%. It indicates that our proposed method can effectively detect small target vehicles and track multi-vehicles in real-time and efficiently, which has a certain promotion effect on promoting in-depth research in the vehicle detection and tracking field.

## Figures and Tables

**Table 1:** The influence of network structure.

Model	Layer	Size	FPS	mAP
YOLOv5s	213	13.7 M	55	0.503
YOLOv5-NAM	219	13.9 M	54.6	0.512

**Table 2:** The influence of loss function.

Model	Loss	Layer	FPS	mAP
YOLOv5s	GIoU	213	55	0.503
YOLOv5s	CIoU	213	55	0.505
YOLOv5-NAM	GIoU	219	54.6	0.512
YOLOv5-NAM	CIoU	219	54.6	0.515

**Table 3:** The influence of NMS method.

Model	NMS	FPS	mAP
YOLOv5s	IoU-NMS	55	0.503
YOLOv5s	SD-NMS	52	0.505
YOLOv5-NAM	IoU-NMS	54.6	0.512
YOLOv5-NAM	SD-NMS	51.8	0.514

**Table 4:** The detection results on UA-DETRAC dataset.

Model	NMS	Cars	Buses	Vans	Other	mAP
YOLOv5s	IoU-NMS	0.697	0.433	0.743	0.139	0.503
YOLOv5-NAM	IoU-NMS	0.7	0.46	0.731	0.175	0.517
YOLOv5s	SD-NMS	0.712	0.424	0.735	0.143	0.504
YOLOv5-NAM	SD-NMS	0.72	0.446	0.726	0.186	0.519

**Table 5:** Detection results on the COCO dataset.

Model	Backbone	AP	${\mathrm{AP}}_{50}$	${\mathrm{AP}}_{75}$	${\mathrm{AP}}_{S}$	${\mathrm{AP}}_{M}$	${\mathrm{AP}}_{L}$
Faster R-CNN [23]	VGG-16	0.219	0.427	–	–	–	–
Mask R-CNN [24]	R-101-FPN	0.382	0.603	0.417	0.201	0.411	0.502
R-FCN [25]	R-101	0.299	0.519	–	0.108	0.328	0.450
SSD [21]	VGG-16	0.288	0.485	0.303	0.109	0.318	0.435
YOLOv3 [28]	Darknet-53	0.33	–	–	–	–	–
RetinaNet [17]	X-101-FPN	0.390	0.594	0.417	0.226	0.434	0.509
FCOS [42]	X-101-FPN	0.421	0.621	0.452	0.256	0.449	0.520
CenterNet [43]	HG-104	0.421	0.611	0.459	0.241	0.455	0.528
YOLOv4 [29]	CSP	0.435	0.657	0.473	0.267	0.467	0.533
YOLOv5s [30]	CSP	0.355	0.55	–	–	–	–
YOLOv5x [30]	CSP	0.472	0.666	–	–	–	–
Ours	CSP-NAM	0.367	0.561	0.378	0.231	0.424	0.492

**Table 6:** The tracking results on UA-DETRAC dataset.

Method	MOTA	MOTP	MT	ML	IDs	FPS
SORT [32]	0.302	0.371	0.178	0.245	868.8	287.6
DeepSORT [33]	0.302	0.369	0.298	0.202	512.7	25.6
JDE [34]	0.334	0.387	0.345	0.156	489.9	66.7
Ours	0.343	0.386	0.367	0.143	414.8	64.6

## Data Availability

The data used to support the findings of this study are included within the article.
